# Obesity Is Underestimated Using Body Mass Index and Waist-Hip Ratio in Long-Term Adult Survivors of Childhood Cancer

**DOI:** 10.1371/journal.pone.0043269

**Published:** 2012-08-14

**Authors:** Karin Blijdorp, Marry M. van den Heuvel-Eibrink, Rob Pieters, Annemieke M. Boot, Patric J. D. Delhanty, Aart-Jan van der Lely, Sebastian J. C. M. M. Neggers

**Affiliations:** 1 Department of Pediatric Oncology/Hematology, Erasmus MC-Sophia Children’s Hospital, Rotterdam, The Netherlands; 2 Department of Medicine – Section Endocrinology, Erasmus University Medical Center, Rotterdam, The Netherlands; 3 Department of Pediatric Endocrinology, University Medical Center Groningen (UMCG), Groningen, The Netherlands; 4 Department of Internal Medicine, Erasmus MC, Rotterdam, The Netherlands; University of Porto, Portugal

## Abstract

**Objective:**

Obesity, represented by high body mass index (BMI), is a major complication after treatment for childhood cancer. However, it has been shown that high total fat percentage and low lean body mass are more reliable predictors of cardiovascular morbidity. In this study longitudinal changes of BMI and body composition, as well as the value of BMI and waist-hip ratio representing obesity, were evaluated in adult childhood cancer survivors.

**Methods:**

Data from 410 survivors who had visited the late effects clinic twice were analyzed. Median follow-up time was 16 years (interquartile range 11–21) and time between visits was 3.2 years (2.9–3.6). BMI was measured and body composition was assessed by dual X-ray absorptiometry (DXA, Lunar Prodigy; available twice in 182 survivors). Data were compared with healthy Dutch references and calculated as standard deviation scores (SDS). BMI, waist-hip ratio and total fat percentage were evaluated cross-sectionally in 422 survivors, in who at least one DXA scan was assessed.

**Results:**

BMI was significantly higher in women, without significant change over time. In men BMI changed significantly with time (ΔSDS = 0.19, P<0.001). Percentage fat was significantly higher than references in all survivors, with the highest SDS after cranial radiotherapy (CRT) (mean SDS 1.73 in men, 1.48 in women, *P*<0.001). Only in men, increase in total fat percentage was significantly higher than references (ΔSDS = 0.22, *P*<0.001). Using total fat percentage as the gold standard, 65% of female and 42% of male survivors were misclassified as non-obese using BMI. Misclassification of obesity using waist-hip ratio was 40% in women and 24% in men.

**Conclusions:**

Sixteen years after treatment for childhood cancer, the increase in BMI and total fat percentage was significantly greater than expected, especially after CRT. This is important as we could show that obesity was grossly underestimated using BMI and waist-hip ratio.

## Introduction

Childhood cancer survival rates have increased enormously over the last few decades [1], [2]. As a consequence, the incidence of treatment related complications is increasing. One of the major sequelae is obesity, with a prevalence of 9 to 30% depending on former treatment modalities [3], [4], [5]. Body mass index (BMI) is the most widely used measure for obesity. However, it has been shown that a high amount of total body fat, high intra-abdominal fat percentage and low lean body mass are more reliable determinants than high BMI in predicting the development of cardiovascular disease or diabetes mellitus [6], [7], [8], [9], [10]. In a recent study, Shah and Braverman showed that BMI misclassified 48% of the female and 25% of the male population using total fat percentage measured by dual X-ray absorptiometry (DXA) as the gold standard. This lead to an underestimation of the prevalence of obesity [10]. Waist circumference, which approximates the amount of intra-abdominal fat, is a more accurate marker than BMI and is one of the criteria used to define metabolic syndrome [11], [12].

In childhood cancer survivors, abnormal body composition has been thoroughly described and is caused by several factors including damage to the hypothalamus and/or pituitary due to cranial radiotherapy and use of corticosteroids [5], [11], [12], [13], [14], [15]. Most studies that investigated changes in body composition, in addition to BMI, were cross-sectional and included small cohorts and selected subgroups, such as acute lymphoblastic leukemia (ALL) survivors [11], [16], [17], [18]. Currently, long-term survivor studies measuring body composition longitudinally are not available. The Childhood Cancer Survivor Study has reported changes in BMI with a very long-term follow-up of 25 years [19]. The aim of our study was to investigate longitudinal changes in BMI and body composition, and to evaluate the value of BMI and waist-hip ratio as compared with total fat percentage measured by DXA in long-term adult childhood cancer survivors in a single center in the Netherlands.

## Subjects and Methods

### Ethics Statement

The data described in the current retrospective study were obtained during regular visits at the late effects clinic, and clinical investigations were assessed using the standard guidelines for screening late effects after childhood cancer following Good Clinical Practice (GCP). An official written informed consent from every patient that visited the outpatient clinic was obtained according to standards of the Institutional Review Board (IRB).

### Subjects

**Figure 1:**
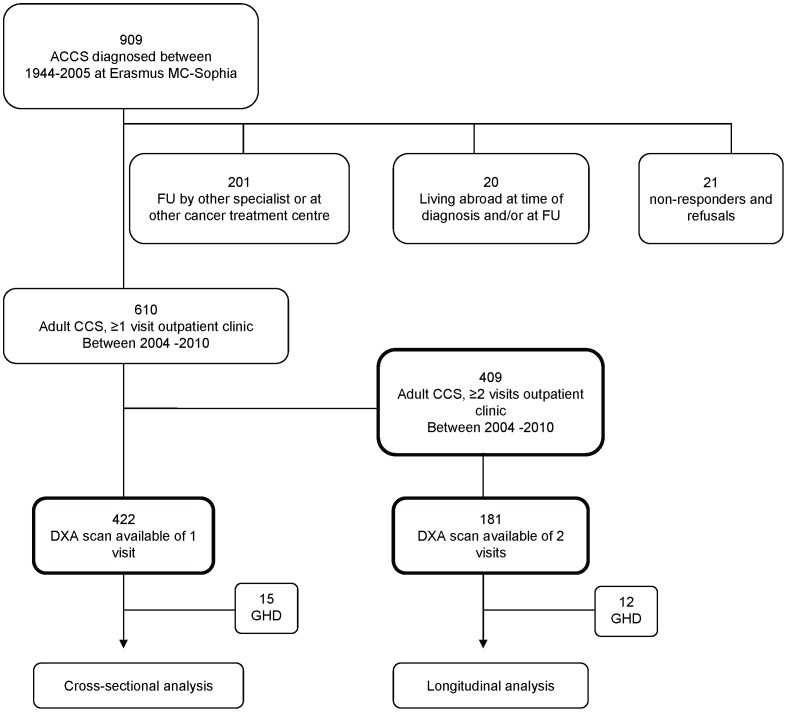
Flowchart of included survivors. **FU** follow-up; **CCS** childhood cancer survivors; **GHD** Growth hormone deficiency.

We performed a retrospective single center study. Follow-up of subjects at our late effects outpatient clinic for long-term childhood cancer survivors starts 5 years after cessation of therapy and is individualized based on cancer diagnosis and treatment protocol. Out of 909 adult childhood cancer survivors diagnosed and treated between 1964 and 2005, 610 had visited the outpatient clinic and 410 had visited the outpatient clinic twice (figure 1). Longitudinal changes in BMI were evaluated in survivors with 2 visits. Cross-sectional evaluation of BMI, waist-hip ratio and total fat percentage was assessed in 423 survivors. One survivor was diagnosed with Down syndrome and was therefore excluded.

### Methods

Treatment details, as well as disease and patient characteristics, were retrieved from our local database (table 1). Information regarding pituitary dysfunction and hormone supplementation were evaluated. Body mass index (BMI) was calculated from height and weight [20]. Waist-hip ratio was calculated from waist and hip circumference [21]. Lean body mass and total fat percentages were measured by DXA using a single machine (DXA, Lunar Prodigy, GE Healthcare, Madison, WI, USA). Data from sequential DXA scans was available in 182 survivors. Obesity was defined as BMI ≥30 [22], total fat percentage ≥25% (M) and ≥30% (W) [23] and waist-hip ratio ≥0.85 (W) and ≥0.90 (M) [21].

Final height data were compared with Dutch adult references described by Fredriks *et al.* [24]. BMI data were compared with data from self-reported questionnaires, which are sent out yearly to 10 000 subjects by the central office for statistics (CBS) in the Netherlands [25]. Lean body mass and total fat percentage data were compared with healthy Dutch references [26], [27]. To adjust for age and gender, standard deviation scores were calculated for final height, BMI and body composition measures.

Growth hormone deficiency (GHD) was defined by an insufficient growth hormone stimulation test (growth hormone peak <3 µg/L during an insulin tolerance test or <9 µg/L during a GHRH-Arginine test [28]). Hypothyroidism was defined as an fT4 level <11 pmol/L in combination with normal or high TSH levels (>4.3 mU/L; primary hypothyroidism) or a TSH level <0.4 mU/L (secondary hypothyroidism). Hypogonadism was defined as a testosterone level <9 nmol/L in men or oligo/amenorrhea in women.

### Laboratory Measurements

Serum insulin-like growth factor-I (IGF-I; nmol/L) was measured using a chemi-luminescence-based immunoassay (Immulite 2000, Siemens DPC, Los Angeles CA, USA). Intra- and interassay coefficients of variation (CV) were <5 and <7%. IGF-I levels were compared with reference values by using standard deviation scores (SDS) [29]. In cases where GHD was suspected (i.e. IGF-I below −2 SDS) a growth hormone stimulation test was assessed. Testosterone (nmol/l) was measured by coated tube radioimmuno-assay (Siemens DPC). Intra- and interassay CVs were <6 and <9% respectively. Thyroid stimulating hormone (TSH) (U/L) and free thyroxine (fT4) (pmol/l) were measured using chemoluminescence assays (Vitros ECi Immunodiagnostic System; Ortho Clinical Diagnostics, Rochester, NY). Interassay CV was 4.7–5.4% for fT4 and 2.5–4.1% for TSH.

### Statistics

**Figure 2:**
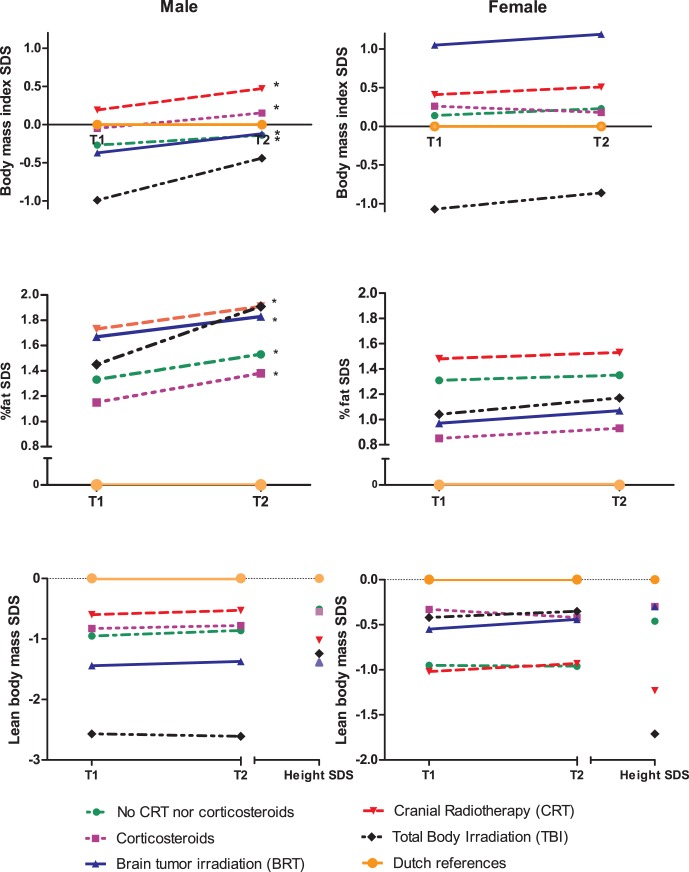
Standard deviation scores (SDS) of body mass index, total fat percentage, lean body mass and height in different therapy groups expressed as mean at T1 and T2. * Significantly higher increase as compared to references (one-paired T-test, p<0.05).

To evaluate longitudinal changes in BMI and body composition, treatment modalities were categorized into five groups: cranial radiotherapy as defined by central nervous system irradiation in leukemia/lymphoma survivors (CRT), brain tumor irradiation directed to the indicated tumor field (BRT), total body irradiation (TBI), corticosteroids (without irradiation), and other treatment (neither cranial irradiation nor corticosteroids). Standard deviation scores were calculated for BMI, total fat percentage and lean body mass and were compared with healthy references using the one sample t-test. When analyzing the rough data, differences between 1^st^ and 2^nd^ assessments were tested using the paired t-test (figure 2). Growth hormone deficient subjects were excluded from the analysis because growth hormone treatment causes a significant decrease of total body fat [30]. Multivariate regression analysis was performed to determine the influence of age at diagnosis, hypothyroidism, hypogonadism and use of oral contraceptives on BMI and measures of body composition.

To evaluate the value of BMI and waist-hip ratio, total fat percentage, retrieved from DXA scans, was used as the gold standard. Total fat percentage was compared with BMI and waist-hip ratio, to determine percent agreement and disagreement, separately in men and women. A Receiver Operating Curve (ROC) analysis stratified by sex was used to evaluate sensitivity and specificity for BMI and waist-hip ratio and to determine more accurate cut-off points relative to total fat percentage. Survivors who had been treated with abdominal radiotherapy were excluded from the waist-hip ratio analysis.

P-values were considered statistically significant if p<0.05. Statistical analysis was performed using SPSS 17.0 software (SPSS, Chicago, IL).

## Results

### Survivors

Baseline and treatment characteristics are shown in Table 1. Median follow-up time between cessation of therapy and the first visit at the outpatient late effects clinic was 16 years (interquartile range (IQR) 11–21). Median time between the 1st assessment (defined as T1) and 2nd assessment (defined as T2) was 3.2 years (IQR 2.9–3.6). Forty-five, mainly leukemia survivors, had been treated with CRT (25 Gy (24–25)), including 3 survivors that had received craniospinal irradiation; thirty-nine, mainly brain tumor survivors, had been treated with BRT (40 Gy (35–44)). Two leukemia survivors had been treated with CRT and TBI and were analyzed in the CRT group. One brain tumor survivor had received BRT and TBI and was analyzed in the BRT group. One hundred and forty-three survivors had been treated with corticosteroids but not cranial irradiation, and 170 had only been treated with chemotherapy, not with corticosteroids or cranial irradiation.

**Table 1:** Baseline characteristics of survivors included in this study.

	Survivors with DXA scan *(cross-sectional data)*		Survivors with two visits *(longitudinal data)*	
	Male N = 243	Female N = 179	Male *N* = 229	Female *N* = 180
Age at diagnosis (yrs)	6.3 (3.4–11.8)	6.5 (3.2–11.7)	6.7 (3.3–11.8)	6.2 (2.9–11.7)
Age at follow-up (yrs)	25.8 (21.9–31.4)	26.6 (22.3–32.7)	23.8 (20.2–28.0)	25.4 (21.0–30.1)
Time between DXA’s (yrs)	n.a.	n.a.	3.2 (2.9–3.6)	3.1 (2.9–3.4)
Follow-up time (yrs)	17.5 (12.1–23.0)	17.9 (13.2–23.7)	15.9 (11.2–20.3)	17.0 (11.9–22.4)
*Diagnosis*				
ALL, T-NHL	107 (44)	82 (46)	79 (35)	58 (33)
AML	10 (4)	7 (4)	8 (4)	8 (4)
B-NHL	28 (12)	14 (8)	24 (11)	14 (8)
Hodgkin lymphoma	32 (13)	14 (8)	25 (11)	16 (9)
Bone tumor	4 (2)	6 (3)	11 (5)	8 (4)
Wilms tumor	26 (11)	21 (12)	26 (11)	24 (13)
Neuroblastoma	5 (2)	10 (6)	11 (5)	18 (10)
Germ cell tumor	1 (1)	1 (1)	4 (2)	5 (3)
MMT	6 (3)	5 (3)	17 (7)	13 (7)
Brain tumor	15 (6)	9 (5)	15 (7)	7 (4)
Other	17 (7)	16 (9)	9 (4)	9 (5)
*Therapy*				
CRT	43 (18)	28 (16)	26 (11)	19 (11)
BRT	9 (4)	3 (2)	31 (14)	8 (4)
Corticosteroids	124 (51)	88 (49)	75 (33)	68 (38)
No CRT/corticosteroids	54 (22)	50 (28)	91 (40)	79 (44)
TBI	13 (5)	10 (6)	6 (3)	6 (3)
Abdominal RT	15 (6)	14 (8)	n.a.	n.a.

Data are expressed as median (interquartile range) or as frequencies (N %).

**ALL** Acute lymphoblastic leukemia; **AML** Acute myeloid leukemia; **B-NHL** B-cell non hodgkin lymphoma; **T-NHL** T-cell non hodgkin lymphoma; **MMT** Malignant mesenchymal tumor; **CRT** Cranial radiotherapy; **BRT** Local radiotherapy on cranium; **TBI** Total body irradiation.

Fifteen survivors had been diagnosed with GHD earlier and were treated with growth hormone (GH) therapy at time of follow-up, and were excluded from further analysis. Hypothyroidism was present in 14 survivors at T1 and 18 survivors at T2. Hypogonadism was present in 21 survivors at T1 and 25 survivors at T2. There were no subjects with untreated hypogonadism and hypothyroidism. Oral contraceptives were used by 94 women at T1 and 86 at T2. Multivariate analysis showed that total fat percentage was not associated with hypogonadism or hypothyroidism during adequate replacement therapy, nor with oral contraceptive use. After correction for height SDS, lean body mass was significantly associated with hypothyroidism in female survivors, which were therefore excluded from the analysis (β −1.20, p = 0.039). BMI was significantly associated with hypogonadism in male survivors, which were therefore excluded from the analysis (β 0.92, p = 0.015). Age at diagnosis was not significantly associated with BMI and body composition.

### Body Mass Index

Male survivors had a significantly lower BMI at T1 (SDS =  −0.17, P = 0.022) and showed a significant change over time (ΔSDS  = 0.19, P<0.001). When analyzing treatment groups separately BMI at T1 was not significantly different from Dutch references, but changed significantly over time in all groups except for TBI (figure 2). In female survivors BMI was significantly higher compared with references, without significant change over time (SDS T1 = 0.22, P = 0.024, T2 = 0.25, P = 0.006). When analyzing treatment groups separately only cranial irradiated female leukemia/lymphoma survivors had significantly higher BMI without significant change over time (SDS T1 = 0.41, P = 0.021, T2 0.51, P = 0.031). Both male and female TBI survivors had a significantly lower BMI compared with references (figure 2).

### Total Fat Percentage

Fat percentage was significantly higher compared with Dutch references in both men (SDS T1 1.37, *P*<0.001) and women (SDS T1 1.05, *P*<0.001). The highest fat percentage was found after CRT (mean SDS T1 1.73 in men, 1.48 in women, *P*<0.001) (figure 2). In men, fat percentage increased significantly (ΔSDS = 0.22, *P*<0.001). Evaluating treatment groups separately, fat percentage increased significantly in all groups except for the TBI group (figure 2). In female survivors no significant increase of fat percentage was found (ΔSDS = 0.07, *P* = 0.22).

### Height and Lean Body Mass

Lean body mass, as well as height, were significantly lower in all treatment groups, compared with Dutch references, except for female BRT survivors (figure 2). Adjusted for height SDS, lean body mass SDS was significantly lower in male TBI survivors only (T1, *P* = 0.01; T2, *P* = 0.004). Lean body mass did not change over time in either men or women in any of the treatment groups (figure 2).

### BMI and Waist-hip Ratio as Compared with Total Fat Percentage

**Figure 3:**
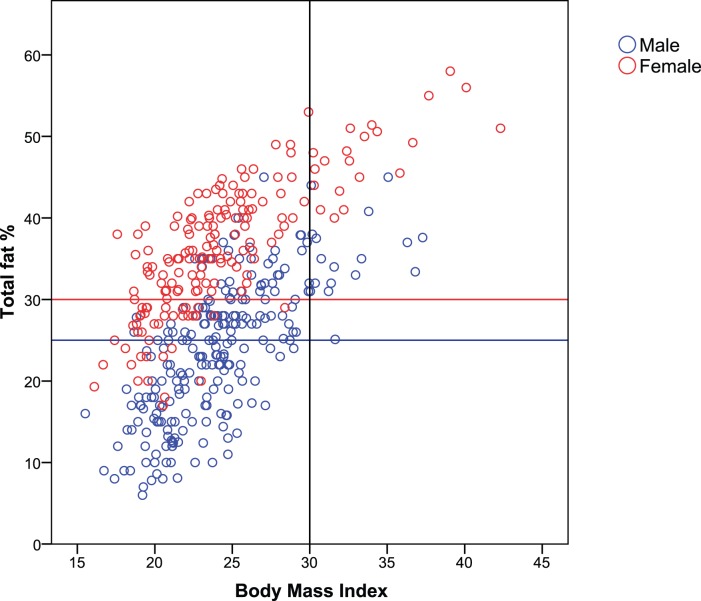
BMI versus total fat percentage. Female and male survivors in the upper quadrant corner, marked by the red respectively the blue horizontal line and black vertical line, are classified as non-obese by BMI, but as obese by total fat percentage.

**Figure 4:**
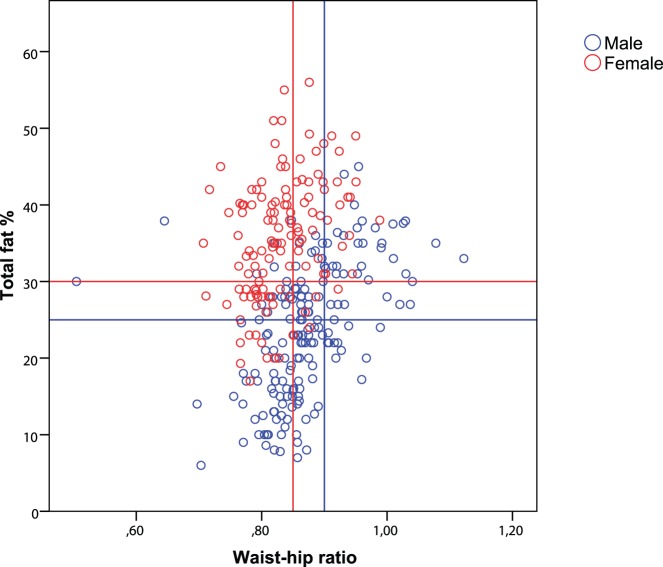
Waist-hip ratio versus total fat percentage. Female and male survivors in the upper quadrant corner, marked by the red respectively the blue lines, are classified as non-obese by waist-hip ratio, but as obese by total fat percentage.

Out of 407 survivors 210 (52%) were misclassified as non-obese based on BMI, while meeting the obesity criteria based on total fat percentage. In female survivors this percentage of misclassification was even higher (65%) than in men (42%) (Table 2, figure 3). Waist-hip ratio was classified as non-obese and total fat percentage as obese in 92 out of 299 survivors (31%). This percentage of misclassification was 40% in female and 24% in male survivors (figure 4). We attempted to define new cut-off points for BMI and waist-hip ratio to better identify childhood cancer survivors as obese, using total fat percentage as the gold standard (table 3). Sensitivity improved by 58% (from 14% to 72%) in males, with a loss in specificity of only 24% (100% to 76%) using a BMI cut-off of 24 instead of 30. In females a cut-off of 22 would give an increase in sensitivity of 64% (16 to 80%) with a loss in specificity of only 18% (100% to 82%). Cut-off points for waist-hip ratio, as defined by the WHO, were more accurate than BMI in childhood cancer survivors (table 3), although a cut-off limit of 0.82 instead of 0.85 in women would give an increase in sensitivity of 26% (45% to 71%), with a loss in specificity of 14% (85% to 71%).

**Table 2:** Total fat percentage, BMI and waist-hip ratio for adult survivors of childhood cancer.

	Body Mass Index			Waist-hip Ratio*		
	Men N = 235	Women N = 172	Total N = 407	Men N = 174	Women N = 125	Total N = 299
**Concordant**						
BMI/WHR non obese, total fat % non-obese	120 (51)	39 (23)	159 (39)	80 (46)	29 (23)	109 (37)
BMI/WHR obese, total fat % obese	17 (7)	21 (12)	38 (9)	40 (23)	41 (33)	81 (27)
**Discordant**						
BMI/WHR non-obese, total fat % obese	98 (42)	112 (65)	210 (52)	42 (24)	50 (40)	92 (31)
BMI/WHR obese, total fat % non-obese	0	0	0	12 (7)	5 (4)	17 (6)

* Survivors treated with abdominal radiotherapy were excluded.

**Table 3:** Sensitivity and specificity for different cut-off points of BMI and waist-hip ratio as defined by total fat percentage of ≥25% for men and ≥30% for women.

	Male		Female	
	Sensitivity	Specificity	Sensitivity	Specificity
**BMI cut-off point**				
18	100	4	99	10
20	98	25	92	54
22	91	58	80	82
24	72	76	53	97
26	47	95	32	97
28	28	98	23	97
**30** *	**14**	**100**	**16**	**100**
32	6	100	11	100
**WHR cut-off point**				
0.77	98	3	93	9
0.78	98	8	89	21
0.79	98	9	84	32
0.80	96	12	80	50
0.81	94	15	75	59
0.82	91	22	71	71
0.83	88	34	60	76
0.84	86	42	54	82
**0.85** *	81	48	**45**	**85**
0.86	73	56	42	88
0.87	63	70	33	88
0.88	59	76	31	91
0.89	54	85	24	94
**0.90** *	**49**	**87**	20	97
0.91	42	88	13	97
0.92	40	91	12	97
0.93	33	95	9	100
0.94	28	96	n.a.	n.a.
0.95	28	97	n.a.	n.a.
0.96	22	97	n.a.	n.a.
0.97	19	98	n.a.	n.a.
0.98	17	99	n.a.	n.a.

* Cut-off limits of BMI respectively waist-hip ratio generally used for the classification of obesity.

## Discussion

Male survivors showed an increase of BMI and total fat percentage during a median follow-up time of 3.2 years, more than 15 years after treatment for childhood cancer. Female survivors, especially the cranial irradiated leukemia/lymphoma subgroup, showed higher BMI and total fat percentage at both time points but without a significant change over time. To our knowledge, the childhood cancer survivor study (CCSS) is the only study to date that describes longitudinal changes in childhood cancer survivors at very long-term follow-up [19]. In this study, self-reported BMI was evaluated in ALL survivors 25 years after diagnosis, and CRT was defined as a risk factor for BMI change, particularly in women [19]. Brouwer *et al.* showed that BMI increased in Dutch childhood cancer survivors, from the end of treatment until attainment of final height and observed that high dose CRT was the most important risk factor for this BMI increase [31]. Long after attainment of final height, all of our male survivors showed a higher BMI increase compared with the reference population, suggesting that cancer treatment effects persist over time. This BMI increase did not result in a significantly higher BMI 3 years later. However, taking into account the short time-interval, this significant increase in BMI at a relatively young age might indicate an emerging problem in the future. In female survivors BMI was significantly higher after CRT treatment; however, there was no significant change over time, suggesting that changes in BMI had already occurred at a younger age. Furthermore, the interval between measurements was only 3 years and survivors had a younger age at the 1^st^ assessment (median 24 years) as compared with the CCSS (median 32 years) which might explain the different findings. Additionally, since annual reference data from the CBS are self-reported, BMI could be underestimated, possibly affecting the comparison between BMI in survivors and controls in this study.

Previous studies have been mainly based on self-reported or measured BMI values. Recently it was shown that BMI is an inaccurate marker for total body fat. Obesity was underestimated in 39% of subjects, using BMI [10]. Therefore, we used DXA to assess total fat percentage and lean body mass, which are considered to be more relevant predictors for cardiovascular disease or diabetes mellitus [6], [7], [8], [9]. In the current study, total fat percentage was persistently and significantly higher than the reference population and was more pronounced in CRT survivors. Furthermore, while mean BMI standard deviation scores varied around zero, increases in total fat percentage SDS and decreases in lean body mass SDS were more pronounced. This implies that although BMI may seem normal, the distribution of total fat and lean body mass might be dramatically changed. Therefore, in order to test the hypothesis that BMI underestimates the adiposity risk in childhood cancer survivors, BMI data were compared with total fat percentage of all the survivors in whom one DXA scan was performed. This revealed that indeed 42% of male survivors and 65% of female survivors were classified as non-obese by BMI, but as obese by total fat percentage. These percentages of misclassification are even greater than compared with earlier findings in the general population by Shah and Braverman, although our subjects were much younger [10]. This finding emphasizes the need for more precise measurements of total fat percentage in order to define obesity. Since DXA is costly and not easily accessible for less developed countries, we attempted to define more accurate cut-off limits for BMI in childhood cancer survivors. It was shown that a cut-off limit of 24 for males and 22 for females resulted in greater sensitivity, with only small reductions in specificity. Although the group was too small to draw definite conclusions, these findings indicate that the current cut-off of 30 is not useful in defining obesity in childhood cancer survivors. A more reliable determinant than BMI that predicts the development of cardiovascular disease is the amount of intra-abdominal fat, which is approximated by the waist-hip ratio [11], [12]. Above all, waist-hip ratio is an easy to use clinical tool. However, when we compared waist-hip ratio with total fat percentage in childhood cancer survivors, percentage misclassification was lower than with BMI, but still 31%.

Mechanisms that contribute to abnormal body composition in childhood cancer survivors include decrease in physical activity, hypothalamic-, pituitary and/or gonadal damage, due to cranial and abdominal radiotherapy and leading to endocrinopathies and use of corticosteroids, followed by metabolic changes [5], [11], [12], [13], [14], [15].

### Limitations

In the current retrospective study no data concerning socio-economic status, smoking, diet and physical activity for the total cohort of survivors, which could be important predictors for BMI change, were available. Reduced energy expenditure and excess energy intake may play a role in the development of overweight in childhood cancer survivors, and has been described during and shortly after treatment [32], [33]. Furthermore, since obesity is not an independent risk factor for cardiovascular morbidity, prospective studies are needed, investigating multiple risk factors such as hypertension, dyslipidemia, insulin resistance and life style changes in very long-term survivors of childhood cancer.

### Conclusion

In this large and unique longitudinal study among childhood cancer survivors, a greater increase in BMI and total fat percentage compared with the reference population was found, especially in adult male survivors,. Furthermore, we found that BMI underestimated obesity by 52% of adult survivors, while waist-hip ratio underestimated 31% of survivors when compared with measurements of total fat percentage. Therefore, we have suggested new cut-off limits, which need to be confirmed in future prospective studies, in order to define obesity more precisely in childhood cancer survivors.
